# Impact of alternating amino acid sequences on beta-amyloid-induced neurotoxicity and neuroinflammation in Alzheimer's disease

**DOI:** 10.18632/aging.205095

**Published:** 2023-10-10

**Authors:** Zhixin Li, Minqi Peng, Chen Wang, Jiaan Yang, Xiang Li, Jing Zhao

**Affiliations:** 1 State Key Laboratory of Coordination Chemistry, Chemistry and Biomedicine Innovation Center (ChemBIC), School of Chemistry and Chemical Engineering, Nanjing University, Nanjing 210023, China; 2 The Brain Cognition and Brain Disease Institute, Shenzhen Institutes of Advanced Technology, Chinese Academy of Sciences, Shenzhen, Guangdong 518055, China

**Keywords:** alternating amino, beta-amyloid, neurotoxicity, neuroinflammation, Alzheimer’s disease

## Abstract

Alzheimer’s disease (AD) is a chronic neurodegenerative disease and the common cause of dementia. The aggregation of beta-amyloid (Aβ peptide) leading to excessive neuroinflammation is considered to be the neuropathological hallmark of AD, although the precise mechanisms remain unclear. Oligomerization of these peptides may be associated with their 42 amino acid residue arrangement. However, the process of amyloid plaque formation is still not well known. The protein folding-shape code (PFSC) method is a powerful tool to analyze protein confirmation which could exhibit the local structural folding features in detail. In our study, we utilized the PFSC to analyze Aβ peptide in humans and mice and found that mouse Aβ42 is less likely to polymerize than human’s. Subsequently, we used the PFSC method to analyze the 42 amino acids of Aβ, transformed some species in human Aβ42 and obtained 7 mutants. We showed that it was not easy to aggregate Aβ in mutants. Herein, inflammatory responses were decreased, as indicated by the expression of cytokines. We confirmed that the neurotoxicity of mutant human Aβ was decreased by preventing peptide aggregation. This may represent a new therapeutic approach for treating AD.

## INTRODUCTION

Alzheimer’s disease (AD) is a chronic and irreversible neurodegenerative disease characterized by progressive memory impairment and cognitive dysfunction that ultimately leads to dementia, for which there is no cure [1–3]. The main clinical manifestations of AD are extracellular beta-amyloid (Aβ) deposits, neurofibrillary tangles (NFTs) and neuronal dystrophy and loss. The amyloid hypothesis posits that the aggregation of Aβ peptides could form extracellular deposits, which have been considered as the primary cause of AD for decades [4, 5]. Aβ peptides play a central role in AD research, as indicated by molecular genetics and biochemical pathology, due to their involvement in all known forms of familial AD [6].

Aβ peptide is the product of the amyloidogenic cleavage of the larger glycoprotein amyloid precursor protein (APP) by β–secretase or γ–secretase, which generates 36–42-residue Aβ. Aβ42 is the most toxic form of Aβ generated through amyloidogenic processing, which readily aggregates into soluble oligomers and insoluble fibril [7–9]. The cytotoxicity of Aβ soluble oligomers has been confirmed, as they activate microglia and impair synaptic functions, leading to neuronal toxicity [10]. Similar results have also been observed in various transgenic mouse models of AD [3, 11]. NFTs are paired helical filaments (PHFs) formed of hyperphosphorylated tau protein, leading to neurotoxicity [12]. In familial AD, numerous genetic mutations can increase Aβ isoforms or alter the Aβ 40/42 ratio to cause the formation of amyloid plaques and the development of AD [13, 14]. The abnormal polymerization of amyloid protein can activate microglial cells, leading to the secretion of a large number of proinflammatory factors by microglial cells, which easily causes the central nervous system to be in a state of excessive inflammation [15]. Even though inflammation is primarily protective, excessive inflammation can lead to tissue damage and promote disease progression [16]. In summary, the production and aggregation of Aβ are pivotal in the pathogenesis and progression of AD. Therefore, reducing Aβ aggregation may exert a positive effect on the course of this disorder.

In our study, we used an algorithm approach named the protein folding-shape code (PFSC) method, which integrates geometric, morphological, and topological features to provide a comprehensive description of folding shape patterns [17, 18]. According to the PFSC method and Protein Folding Variation Matrix (PFVM), we analyzed 42 amino acids of Aβ with certain folding patterns along the sequence, the specific number and arrangement of which played an important role in the polymerization of Aβ. Thus, we replaced some certain of these sites with other amino acids, and 7 mutants were obtained. The study of these 7 mutants indicates that they do not readily form oligomers under the same conditions. After stimulating the cells by these mutants, we found that the cells can reduce inflammatory response. This finding provides a simple way to develop therapeutic strategies for curing AD.

## RESULTS

### Mouse Aβ has less probability for aggregation than human’s

Wild-type mice are not susceptible to Alzheimer’s disease, and many studies have indicated that wild-type mice are less prone to plaque formation in the brain [19]. We know that the aggregation of Aβ is a major cause of AD, and mouse can also generate Aβ. However, mice Aβ is less likely to form plaques than human’s, which may be the reason that mice have a lower chance of developing AD. Here, we used the PFSC method to analyze the three-dimensional (3D) structure of Aβ protein in humans and mice.

**Figure 1:**
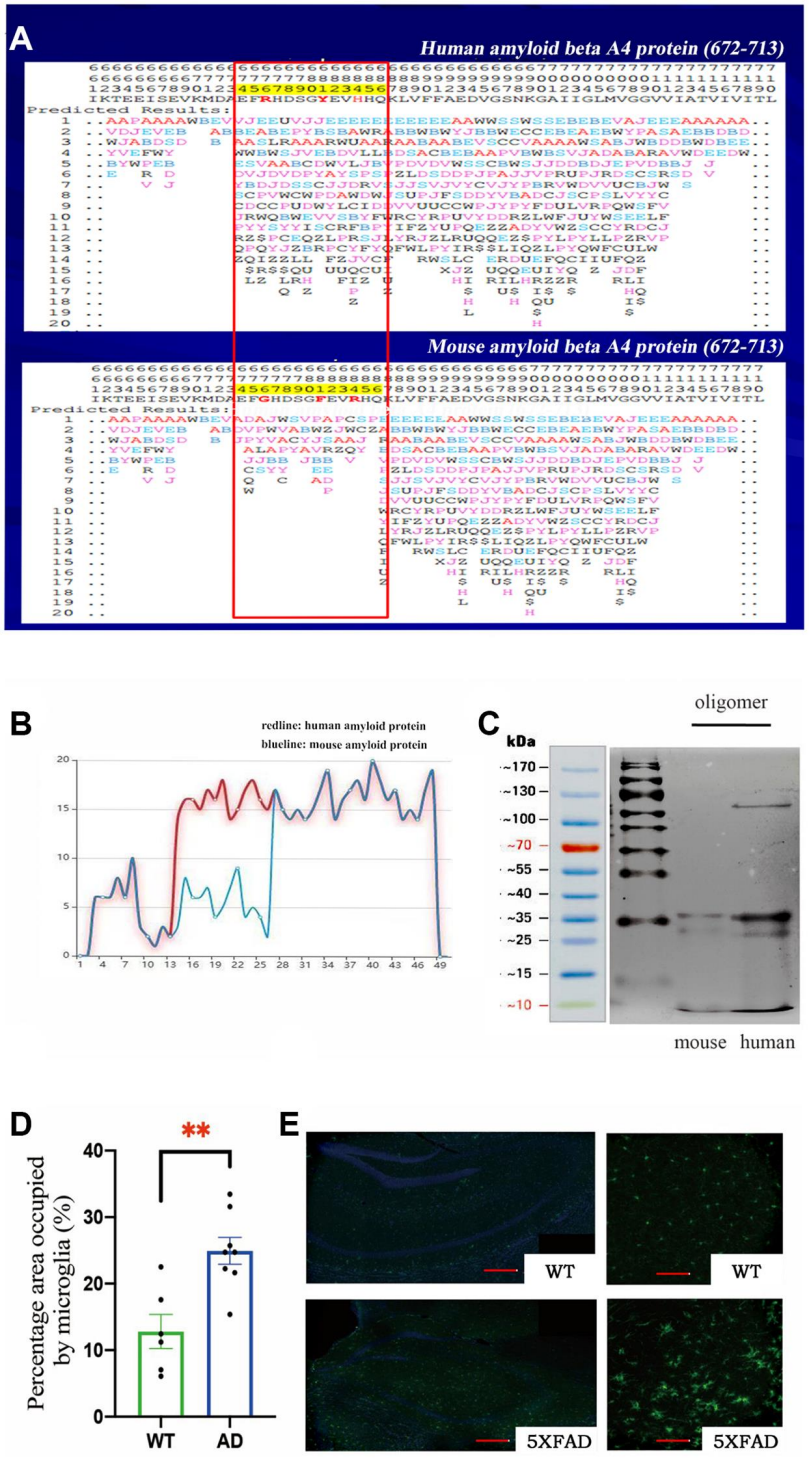
(**A**) The protein conformation fingerprint change matrix of the amino acid sequence of amyloid protein in mice and humans, (**B**) The protein conformation fingerprint change curve of the amino acid sequence of amyloid protein in mice and humans,(**C**) The amyloid protein oligomers in mice and humans, (**D**) The percentage area occupied by microglia in wild-type (*n* = 6) and AD (*n* = 8) samples (^**^P < 0.01), (**E**) The cell bodies of microglia stained with Iba1 antibodies (green) in wild–type and transgenic mice as shown by confocal microscopy. Scale bar = 200 μm for left and 20 μm for right pictures.

We used the PFSC method to predict the protein conformation fingerprint change matrix and the protein conformation fingerprint change curve of the amino acid sequence of amyloid protein in mice and humans. The results are shown in Figure 1A and 1B. First, we utilized the PFSC approach to simulate all feasible polypeptide configurations based on the Aβ sequence of 42 amino acids in humans and mice. Evidently, a higher number of conformational possibilities leads to increase the variability and instability at this site, thereby facilitating polymer formation. It is evident that mouse Aβ displays superior stability compared to its human counterpart, which may account for the reduced propensity towards polymerization. These results suggest that the probability of amyloid polymerization is different in mice and humans. To confirm these results, we used the two amino acid sequences to synthesize amyloid proteins, oligomerized them under the same conditions, and then found that murine Aβ formed fewer oligomers (Figure 1C). These results further explained why normal mice have fewer plaques, since murine Aβ does not polymerize easily. We preliminarily concluded that the different amino acid sequences of Aβ in mice decreased the degree of polymerization. If the mice were given human’s gene, they would develop plaques in their brains and exhibit Alzheimer’s-related symptoms.

We used 5XFAD transgenic mice as the Alzheimer’s disease model in this study. These mice coexpress a total of five FAD mutations (APP K670N/M671L (Swedish) + I716V (Florida) + V717I (London) and PS1 M146L+ L286V). 5XFAD mice have high APP expression and almost exclusively produce Aβ42, which quickly accumulates in the brain. The accumulation of Aβ42 in neurons was observed from 1.5-month-old mice in a mixed genetic background of C57BL/6 and SJL samples. The 5XFAD transgenic mice developed more plaques and produced more oligomers in their brains because they expressed human genes associated with AD.

Microglia are resident innate immune cells of the brain and can be activated by antigens, playing critical roles in the recognition and clearance of Aβ in AD [20]. To study the activation of microglia, we obtained brain tissue from 8-month-old mice and then stained the tissue with Iba1 antibodies to identify microglia. We observed marked activation of microglia and found that the microglial cell body was significantly increased in 5XFAD transgenic mice (Figure 1E). Statistical analysis of the percentage area occupied by microglia confirmed these results (Figure 1D).

### Excessive inflammation in 5XFAD transgenic mice

It is widely accepted that neuroinflammation plays an important role in the progression of AD. Chronic neuroinflammation exerts excessive strain on neurons and eventually leads to neuronal death [21]. 5XFAD mice, which expressed five mutations in human APP and PS1, showed robust Aβ production and obvious plaques as a model of AD. We observed significant activation of microglia in 5XFAD transgenic mice. Microglia are the main trigger of the immune response in the brain. Aβ can bind to microglia, which induces the production of proinflammatory cytokines, such as interferon-γ (IFNγ), tumor necrosis factor α (TNFα), and interleukin-1β (IL-1β), promoting neuroinflammation in AD [22]. There is plentiful evidence that unbridled microglial activity can be harmful to neurons in neurodegenerative diseases [23, 24].

**Figure 2:**
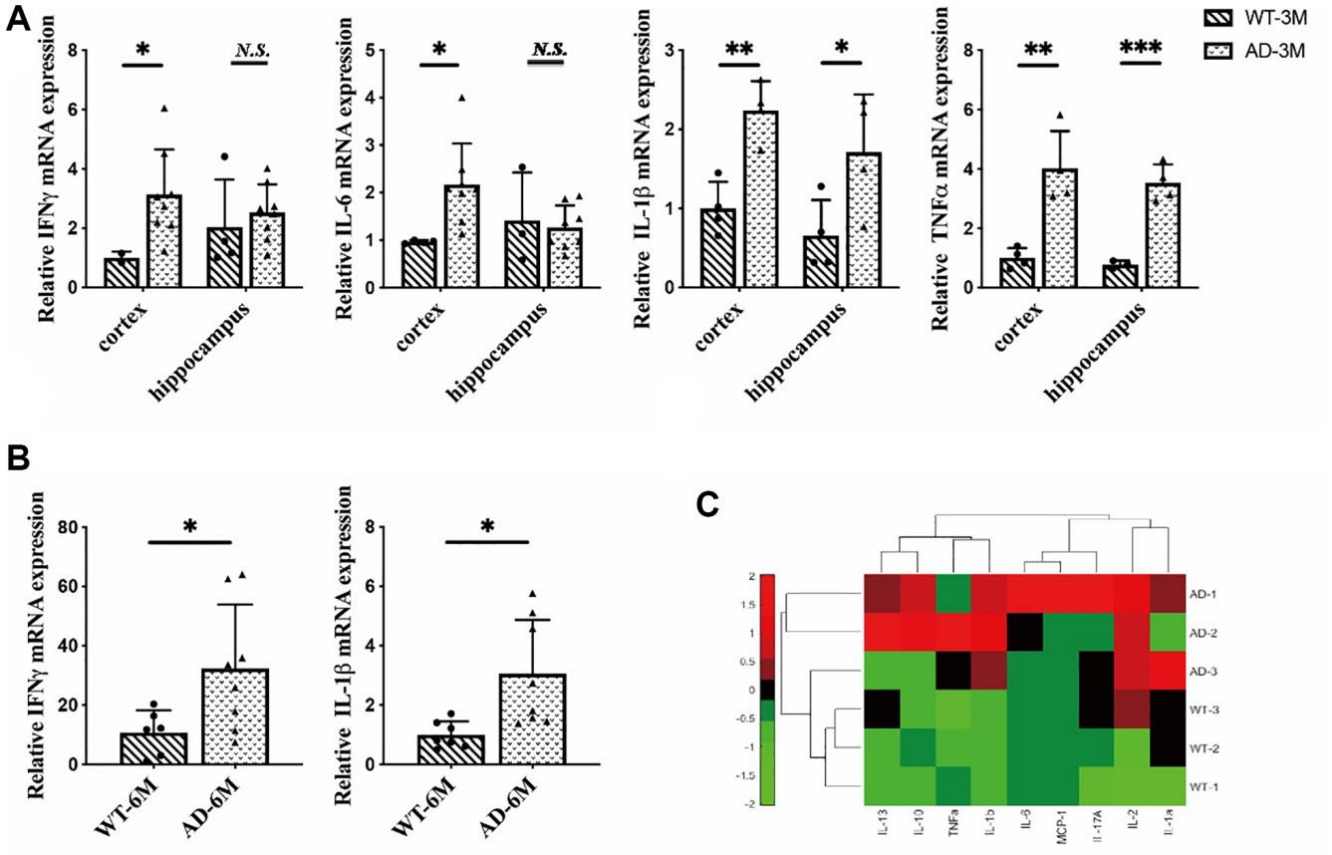
(**A**) The expression of interferon-γ (IFNγ), IL-6, interleukin-1β (IL-1β) and tumor necrosis factorα (TNFα) in the hippocampus and cortex in 3-month-old 5XFAD and wild-type mice (*n* = 4~8), (**B**) The expression of IFN-γ and IL-1β in 6-month-old 5XFAD and wild-type mice (*n* = 7~8), (**C**) The expression of inflammatory cytokines (IL-2, IL-13, IL-10, TNFα, IL-1b, IL-6, MCP-1, IL-17A, IL-1a) in the serum of 8-month-old 5XFAD and wild-type mice (*n* = 3) (^*^P < 0.05, ^**^P < 0.01, ^***^P < 0.001).

Our research showed that the inflammatory response was specifically induced in microglia in the early stages of the disease. First, we focused on inflammation in brain cells. We extracted total RNA from the hippocampus and cortex of 3-month-old mice. In the cortex, we found overexpression of IFNγ, IL-6, IL-1β and TNFα in 5XFAD mice compared to wild-type (WT). In the hippocampus, we also found overexpression of TNFα and IL-1β in 5XFAD mice (Figure 2A), and obtained total RNA from the brains of 6-month-old mice. In addition, we observed the overexpression of proinflammatory cytokines such as interferon-γ and IL-1β in 5XFAD mice (Figure 2B). In summary, the 5XFAD mice showed a significant inflammatory response.

A number of recent studies have demonstrated that peripheral inflammatory conditions can affect the microglial response through neuroimmune communication. If microglia have been activated in AD, being stimulated by peripheral proinflammatory cytokines can amplify this overexpression [25]. To investigate the peripheral inflammatory response in 5XFAD mice serum inflammatory cytokines were measured in 8-month-old mice. The results indicated that inflammatory cytokines were highly secreted in 5XFAD mice (Figure 2C) and verified AD disease symptoms.

### Aβ oligomers stimulate macrophages and microglia to induce inflammatory responses

The Aβ monomer belongs to the class of native IDPs (Intrinsically Disordered Peptides), which aggregate into oligomeric, protofibrils and fibrillar forms that are toxic species that induce neuronal dysfunction [26]. Soluble oligomeric Aβ species are the most cytotoxic forms that can activate microglia to trigger the production of proinflammatory cytokines, ultimately inducing neuronal death [27]. To verify the peripheral immune response in AD, we stimulated macrophages with human Aβ *in vitro*. Macrophages are resident in almost all tissues, and these cells perform a variety of functions throughout the innate immune responses. Macrophages are critical sensor cells that detect infection and induce inflammatory responses by producing cytokines such as TNFα and IL-1β. Therefore, we used Raw 264.7 cells to examine the inflammatory responses stimulated by human Aβ.

**Figure 3:**
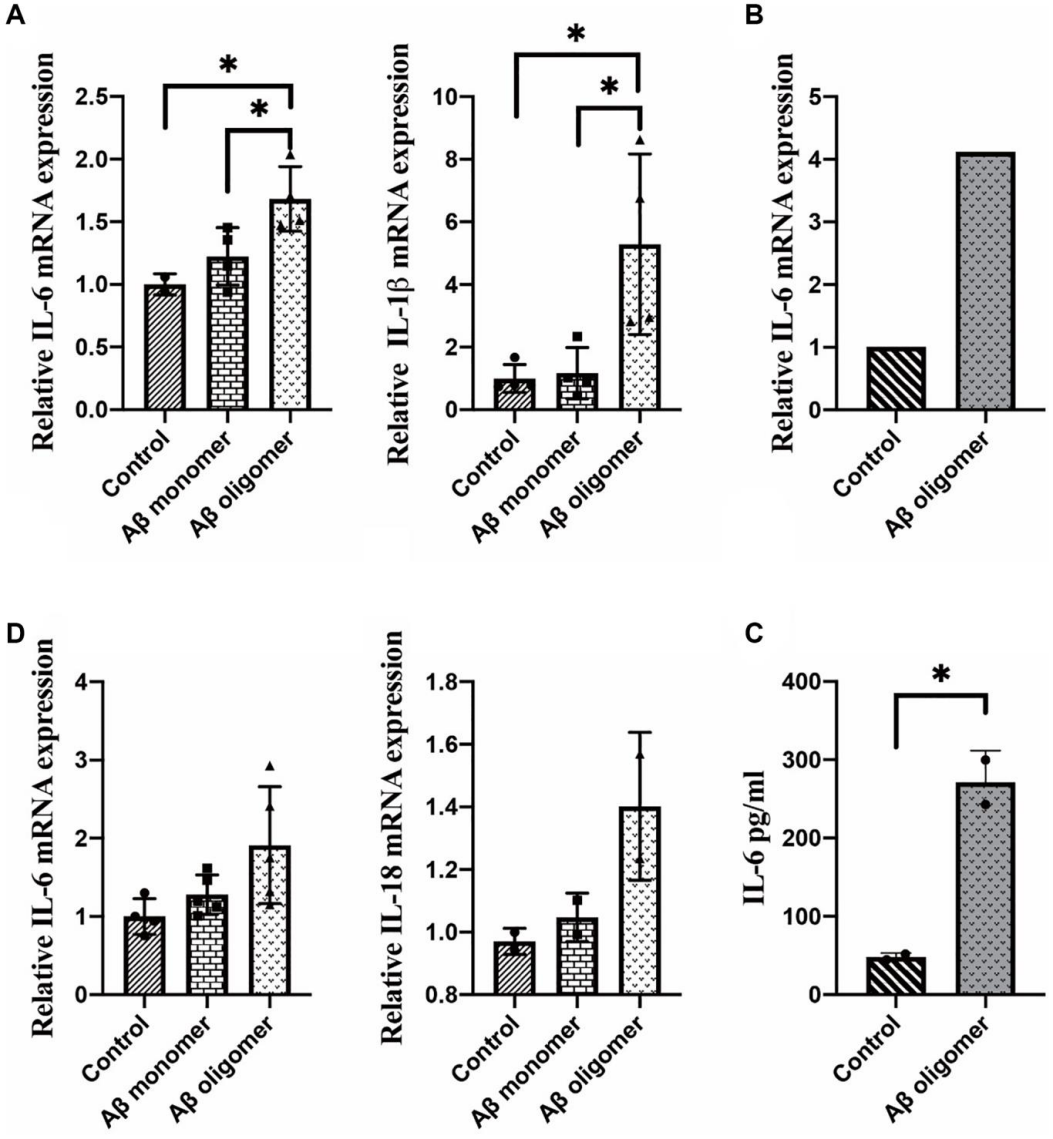
(**A**) The expression of IL-1β and IL-6 after 2 days of stimulation, (**B**) The expression of IL-6 after 1 hour of stimulation, (**C**) The secretion of IL-6 in the culture medium, and (**D**) The inflammatory responses in microglia stimulated by monomers compared with human Aβ oligomers (^*^P < 0.05).

The results showed that Aβ oligomers but not monomers could induce inflammatory responses in macrophages. Overexpression of IL-1β and IL-6 after 2 days of stimulation with Aβ oligomers was found (Figure 3A). We observed overexpression of IL-6 after 1 hour of stimulation with Aβ oligomers (Figure 3B). We also measured the secretion of IL-6 in the culture medium of macrophages with an ELISA kit and obtained consistent results (Figure 3C). In addition, we stimulated microglia with human Aβ *in vitro*. The inflammatory responses could be observed in microglia stimulated by Aβ oligomers but not monomers (Figure 3D).

### Transformed human Aβ is less likely to aggregate and has decreased neurotoxicity

**Figure 4:**
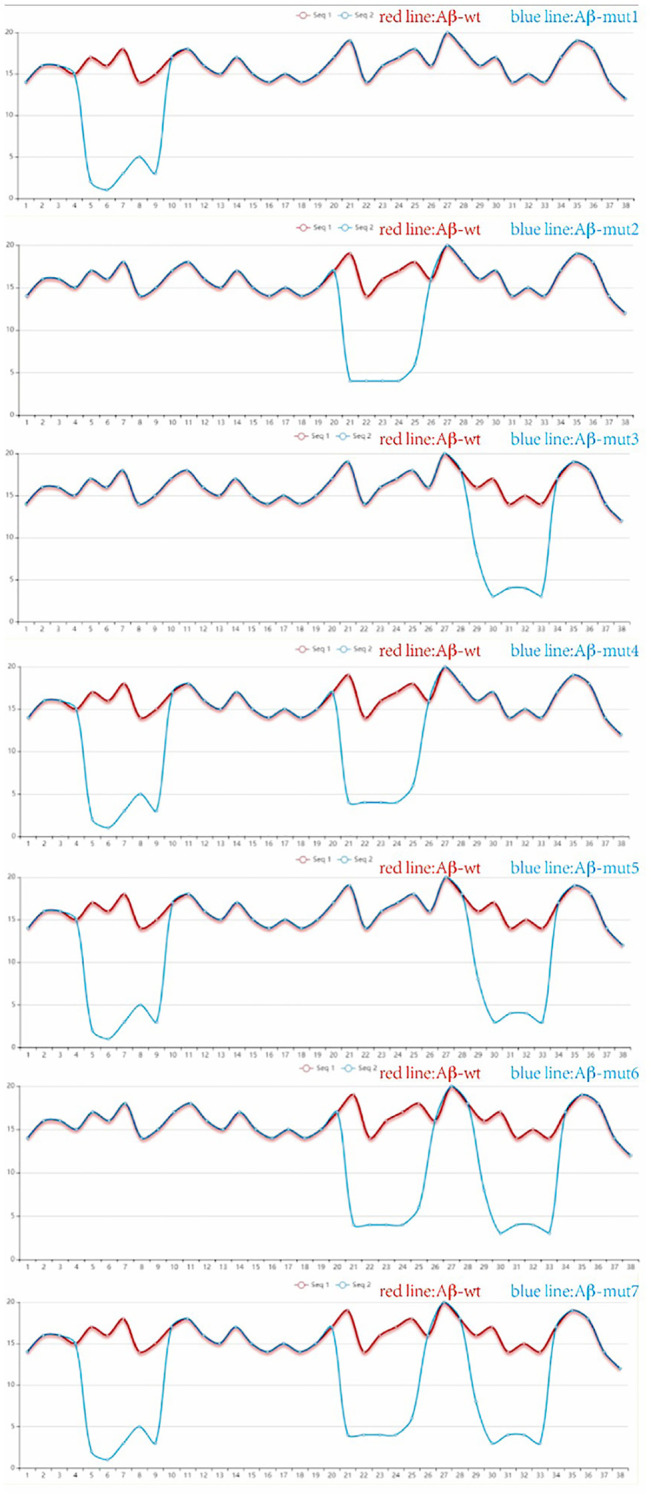
**The protein conformation fingerprint change curves of unmutated compared with seven mutated amino acid sequences.** Five carbon atoms were chosen as a universal folder and all possible folding shapes of local folding variations for all permutation were collected by PFSC method.

Site-directed antibodies that modulate the conformation of the Aβ peptide are the theoretical basis of the immunological approach for the treatment of AD. Changes of the amino acid sequence can directly prevent and reverse protein aggregation in conformational disorders [28]. The aim of this study is to explore the relationship between amino acid sequences, amyloid peptide conformation/aggregation and the signaling cascades responsible for neurotoxicity. Using the PFSC method, we found some certain sites were closely related to polymerization of Aβ42 in humans and amino acid substitutions could reduce the likelihood of Aβ aggregation. The vector characteristics of PFSC in the horizontal and vertical directions were significantly different and these sites were found to be key positions in protein conformation modulation. The way we changed these proteins is shown in Table 1. Protein conformational fingerprint change curves for the unmutated and seven mutated amino acid sequences are shown in Figure 4. Here, we defined the backbone of five amino acid residues was identified as a universal folder. As the X-axis showed, the computational sites were ordered from 1 to 38 in accordance with the amino acid arrangement. The vertical axis of the digital represented all possible folding shapes of local folding variations for all permutation of five amino acids. The results indicated that altering the amino acid sequence from Gly to Ile, Ala and Pro, was an effective strategy for improving the stability of protein structure and reducing the likelihood of forming polymers.

**Table 1:** Amino acid sequences of different types of Aβ.

**Types**	**Amino acid sequences**
Aβ-wt	DAEFRHDSGYEVHHQKLVFFAEDVGSNKGAIIGLMVGGVVIA
Aβ-mut1	DAEFRHDSIYEVHHQKLVFFAEDVGSNKGAIIGLMVGGVVIA
Aβ-mut2	DAEFRHDSGYEVHHQKLVFFAEDVASNKGAIIGLMVGGVVIA
Aβ-mut3	DAEFRHDSGYEVHHQKLVFFAEDVGSNKGAIIPLMVGGVVIA
Aβ-mut4	DAEFRHDSIYEVHHQKLVFFAEDVASNKGAIIGLMVGGVVIA
Aβ-mut5	DAEFRHDSIYEVHHQKLVFFAEDVGSNKGAIIPLMVGGVVIA
Aβ-mut6	DAEFRHDSGYEVHHQKLVFFAEDVASNKGAIIPLMVGGVVIA
Aβ-mut7	DAEFRHDSIYEVHHQKLVFFAEDVASNKGAIIPLMVGGVVIA

**Figure 5:**
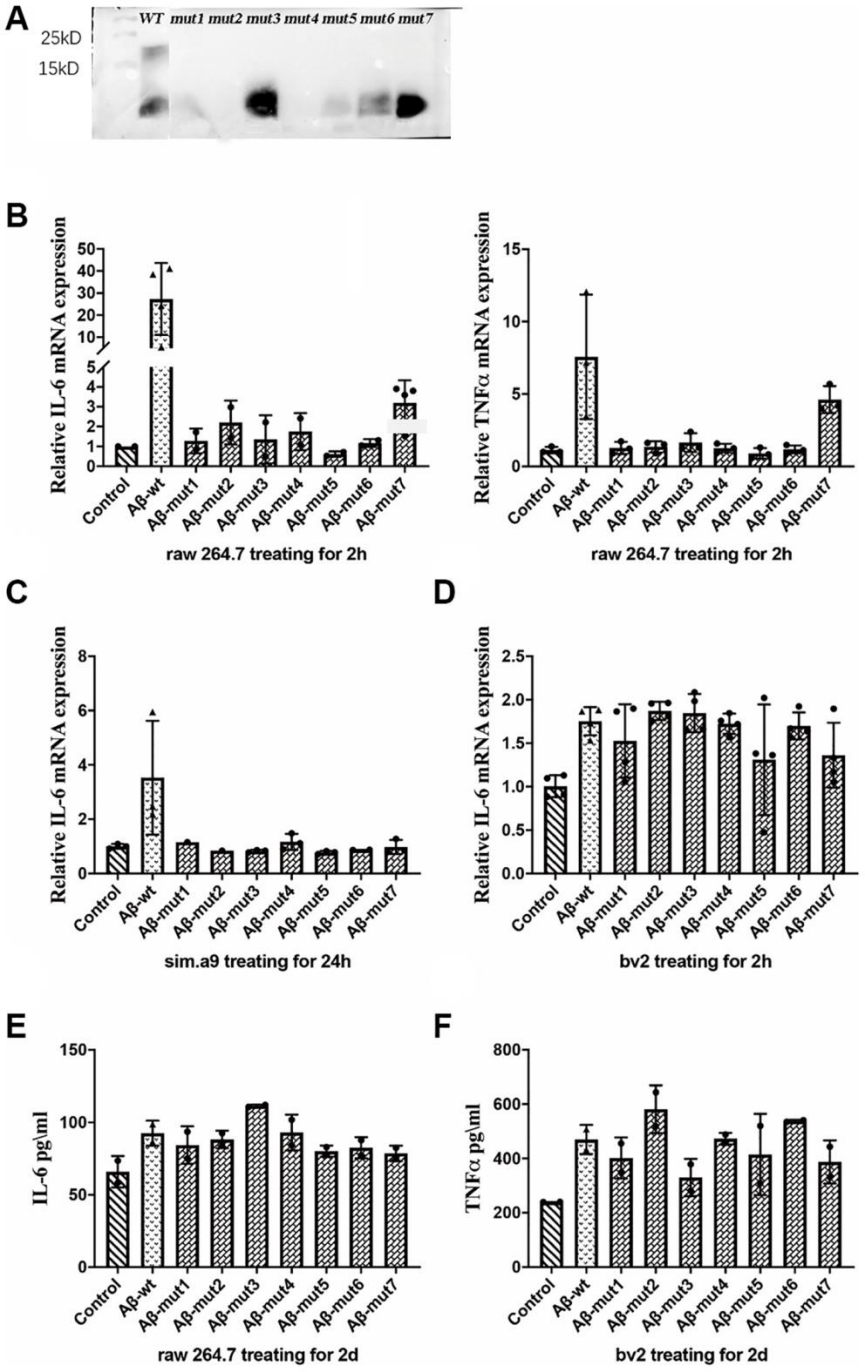
(**A**) Western blot analysis of non-mutants and mutants, (**B**) The expression of IL-6 and TNFα in response to non-mutants and mutants, (**C**, **D**) The inflammatory response of microglial cells to non-mutant and mutants from C57BL/6 mice and bv2 cells respectively, and (**E**, **F**) The concentration of IL-6 in RAW 264.7 cells and TNFα in bv2 cells in response to non-mutant and mutants, as determined by ELISA kits.

After performing the same aggregation procedure *in vitro*, we found reduced aggregation for 7 different types of mutations. We verified the results by Western blot which were shown in Figure 5A. Under normal circumstances, the amino acid polypeptides of humans can form multiple polymers, such as 4-mers, 6-mers and 8-mers. It is evident that the Aβ of mutant individuals could not undergo polymerization during *in vitro* experiments, because the molecular weight of polypeptide was only 4.5KD, similar to that of the Aβ monomer. The Western blot analysis of samples mut1, mut2, and mut4 did not reveal significant bands. This could be attributed to the conformational changes in protein structure caused by amino acid mutations present in these mutant strains, leading to incorrect peptide folding and an increased likelihood of protein degradation.

Nonmutated and mutated Aβ oligomers were used to stimulate cells *in vitro*. The unmutated oligomeric form induced a more robust inflammatory response, whereas the mutated oligomeric and fibrillar conformations were less potent triggers for RAW 264.7 cells. The expression of IL-6 and TNFα was not significantly increased after stimulation with the mutant oligomers (Figure 5B). We also observed a decreased inflammatory response in a microglial cell line (SIM-A9) from C57BL/6 mice (Figure 5C). We confirmed these results in the BV2 murine microglial cell line studies (Figure 5D). After analyzing the secretion of inflammatory factors by stimulated cells, we measured the concentration of IL-6 in the culture medium of RAW 264.7 cells and TNFα in the culture medium with an ELISA kit and obtained consistent results (Figure 5E and 5F).

## CONCLUSION

In our study, we established a probable relationship between the aggregation of Aβ42 and its amino acid sequences. The polymerization of Aβ is the primary etiology of AD and may induce excessive neuroinflammation, which in turn could accelerate the progression of this debilitating disease. We utilized the PFSC method to analyze Aβ42 in humans and mice and showed that the probability of aggregation differed between them. Mouse Aβ42 was less likely to polymerize than human’s, which may be the reason why mice seem resistant to AD. Specifically, mouse Aβ42 exhibits a lower propensity for polymerization relative to its human counterpart, which might account for its resistance to AD. We identified three distinct sites in the amino acid sequences of both humans and mice, and subsequently transformed some of these sites in human Aβ42 to mitigate its propensity for aggregation. This transformation was inspired by the PFSC method, which is commonly employed to analyze modified amino acid sequences. The PFSC method is an algorithmic approach that integrates geometry, morphology, and topology to provide an exhaustive description of proteins. Additionally, the PFSC method is both rigorous and logical in its execution, and its validity has been thoroughly demonstrated. When compared with other methods, all of which relied on a common conclusion for assessing protein structure similarity and complexity. Moreover, we experimentally verified the reliability of this result by employing diverse cell types to confirm our *in vitro* findings. Our observations indicated that the polymerization and neurotoxicity of human Aβ42 were mitigated post-transformation, thereby presenting a potential therapeutic strategy for Alzheimer’s disease.

## MATERIALS AND METHODS

### Animals

Male 5XFAD mice were obtained from Zhaoyan Suzhou New Drug Research Center Co., Ltd. All mice were fed under specific-pathogen-free conditions with a normal day-night schedule and adequate water and food. All procedures were approved by the Institutional Animal Care and Use Committee of Shenzhen Institute of Advanced Technology, Chinese Academy of Sciences, and all experimental procedures on animals were in accordance with the guidelines of the Animal Care and Use Committee.

### Antibodies and immunohistochemistry

For immunohistochemistry, anti-Iba1 (Abcam ab153696) was used to identify microglia in the brain. Alexa Fluor^®^ 488 AffiniPure Donkey Anti-Rabbit IgG (H+L) (Jackson 711-545-152) was used. After being anesthetized, the mice were transcardially perfused with PBS and then with 4% paraformaldehyde (PFA) in PBS (wt/vol). Brains were extracted and fixed at 4°C in 4% PFA overnight and then dehydrated in 30% sucrose in PBS. A freezing microtome was used to cut 40-μm coronal slices. The slices were stained with an antibody in a single well. The slices were incubated overnight at 4°C in primary antibodies and then were incubated for 2 h with secondary antibodies at room temperature. For counterstaining, the slices were incubated for 5 minutes with 4’, 6-diamidino-2-phenylindol (DAPI, 0.4 mg/mL, Sigma). All images were obtained with a Zeiss LSM880 confocal microscope.

### Measurement of inflammatory cytokines

Peripheral blood was drawn from the mice. Centrifugation was performed at 4°C for 20 minutes at 3000 rpm. The supernatant serum was analyzed by a suspension array (Merck Millipore, t-milliplex-m18 h) performed at Mingyan Biological Shenzhen Co., Ltd., China.

200 μl of Wash Buffer was added to each well of a pre-washed 96-well plate, and the plate was shaken at room temperature on an oscillator for 10 minutes. Standard, blank, and sample wells were prepared, including standard wells with different concentrations of the standard, a blank well with standard diluent, and sample wells with corresponding dilutions and test samples. The plate was then incubated at 2-8°C in the dark overnight (16-18 hours). Next, 200 μl of 1x Wash Buffer was added to each well three times. 25 μl of antibody detection reagent was added to each well and the plate was shaken at room temperature for 1 hour in the dark. 25 μL of SAPE was then added to each well and the plate was shaken at room temperature for 30 minutes in the dark. The plate was washed three times with 200 μl of 1x Wash Buffer. Finally, 150 μl of Sheath Fluid or Drive Fluid was added to each well, and the plate was shaken for 5 minutes at room temperature in the dark. Data were analyzed using Milliplex Analyst 5.1 software (EMD Millipore, Billerica, MA, USA).

### Cell culture and stimulation

All cells were routinely cultured in DMEM (Gibco, 11995065) supplemented with 10% fetal bovine serum (Gibco, 10270-106), 100 units/ml penicillin, and 100 μg/ml streptomycin in a 37°C incubator with a humidified atmosphere of 5% CO_2_. The cultured cells were passaged every two days and seeded at a density of approximately 2 × 10^5^/ml. Then, the cells were stimulated with vehicle, 2 μM Aβ (1–42) or mAβ (Aβ monomer).

mAβ was prepared by dissolving Aβ (1–42) peptides (ANASPC, AS-20276) to 5 mM in anhydrous dimethyl sulfoxide (anhydrous DMSO; Sigma-Aldrich, d2650) and used immediately. After ultrasonic processing at room temperature in 10 minutes, oAβ (Aβ oligomer) and fAβ (Aβ fiber) were prepared by diluting the mAβ solution to 100 μM in DMEM F12 (Gibco, 11320033). The resulting solution was then incubated on ice for 24–26 h. Centrifugation was performed at 4°C for 10 minutes at 14,000 rpm. The supernatant contained oAβ, and the sediment contained fAβ.

### Quantification of mRNA levels by real-time PCR

Total RNA was extracted from cell or tissue samples and purified using the TransZol Up Plus RNA Kit (TransGen Biotech, ER501). For mRNA analysis, first-strand cDNA was synthesized using TransScript^®^ One-Step gDNA Removal and cDNA Synthesis SuperMix (TransGen Biotech, AT311). Two microliters of cDNA were used for qRT-PCR, and the reaction was performed at 95°C for 2 min, followed by 40 cycles of 95°C for 10 s and 60°C for 30 s using TransStart^®^ Tip Green qPCR SuperMix (TransGen Biotech, AQ141) with gene-specific primers (Supplementary Table 1). Gene expression levels were normalized to Rps18. The data were analyzed using QuantStudio Design and Analysis software v1.5.1, and gene expression was calculated using the 2^−ΔΔCt^ method.

### The PFSC method

The PFSC method is an algorithmic approach that integrates geometry, morphology and topology to achieve an exhaustive description of protein folding structures, including both regular secondary structures and irregular tertiary structures. It contains 27 vectors expressed by 27 symbols (26 alphabetic letters in uppercase and “$” sign), and each of the letters is used to describe a 3D folding shape of five successive Cα atoms as a basic unit along the protein backbone. The PFSC method uses three components, which are two torsion angles for each of the four adjacent Cα atoms and one pitch distance between two ending Cα atoms from Cα (n−2) to Cα (n+2), to represent the folding shapes of protein structures. For example, the letter “A” represents a typical α-helix and “B” a typical β-strand. Other letters represent various possible folding shapes, including those partially resembling α-helix or β-strand, and those describing irregular folding. Given a 3D protein structure input, a computer program can generate the PFSC as an output to describe the protein folding shape. The accuracy of the PFSC approach has been verified [17].

## Supplementary Materials

Supplementary Table 1

## References

[1] Du X, Wang X, Geng M. Alzheimer's disease hypothesis and related therapies. Transl Neurodegener. 2018; 7:2. 10.1186/s40035-018-0107-y 29423193 PMC5789526

[2] Scheltens P, De Strooper B, Kivipelto M, Holstege H, Chételat G, Teunissen CE, Cummings J, van der Flier WM. Alzheimer's disease. Lancet. 2021; 397:1577–90. 10.1016/S0140-6736(20)32205-4 33667416 PMC8354300

[3] Tu S, Okamoto S, Lipton SA, Xu H. Oligomeric Aβ-induced synaptic dysfunction in Alzheimer's disease. Mol Neurodegener. 2014; 9:48. 10.1186/1750-1326-9-48 25394486 PMC4237769

[4] Burns A, Iliffe S. Alzheimer's disease. BMJ. 2009; 338:b158. 10.1136/bmj.b158 19196745

[5] Jack CR Jr, Knopman DS, Jagust WJ, Petersen RC, Weiner MW, Aisen PS, Shaw LM, Vemuri P, Wiste HJ, Weigand SD, Lesnick TG, Pankratz VS, Donohue MC, Trojanowski JQ. Tracking pathophysiological processes in Alzheimer's disease: an updated hypothetical model of dynamic biomarkers. Lancet Neurol. 2013; 12:207–16. 10.1016/S1474-4422(12)70291-0 23332364 PMC3622225

[6] Selkoe DJ. The genetics and molecular pathology of Alzheimer's disease: roles of amyloid and the presenilins. Neurol Clin. 2000; 18:903–22. 10.1016/s0733-8619(05)70232-2 11072267

[7] Selkoe DJ. Alzheimer's disease: genes, proteins, and therapy. Physiol Rev. 2001; 81:741–66. 10.1152/physrev.2001.81.2.741 11274343

[8] Thinakaran G, Koo EH. Amyloid precursor protein trafficking, processing, and function. J Biol Chem. 2008; 283:29615–9. 10.1074/jbc.R800019200 18650430 PMC2573065

[9] Viswanathan A, Greenberg SM. Cerebral amyloid angiopathy in the elderly. Ann Neurol. 2011; 70:871–80. 10.1002/ana.22516 22190361 PMC4004372

[10] Klyubin I, Betts V, Welzel AT, Blennow K, Zetterberg H, Wallin A, Lemere CA, Cullen WK, Peng Y, Wisniewski T, Selkoe DJ, Anwyl R, Walsh DM, Rowan MJ. Amyloid beta protein dimer-containing human CSF disrupts synaptic plasticity: prevention by systemic passive immunization. J Neurosci. 2008; 28:4231–7. 10.1523/JNEUROSCI.5161-07.2008 18417702 PMC2685151

[11] Kasza Á, Penke B, Frank Z, Bozsó Z, Szegedi V, Hunya Á, Németh K, Kozma G, Fülöp L. Studies for Improving a Rat Model of Alzheimer's Disease: Icv Administration of Well-Characterized β-Amyloid 1-42 Oligomers Induce Dysfunction in Spatial Memory. Molecules. 2017; 22:2007. 10.3390/molecules22112007 29156571 PMC6150403

[12] Fitzpatrick AWP, Falcon B, He S, Murzin AG, Murshudov G, Garringer HJ, Crowther RA, Ghetti B, Goedert M, Scheres SHW. Cryo-EM structures of tau filaments from Alzheimer's disease. Nature. 2017; 547:185–90. 10.1038/nature23002 28678775 PMC5552202

[13] Borchelt DR, Thinakaran G, Eckman CB, Lee MK, Davenport F, Ratovitsky T, Prada CM, Kim G, Seekins S, Yager D, Slunt HH, Wang R, Seeger M, et al. Familial Alzheimer's disease-linked presenilin 1 variants elevate Abeta1-42/1-40 ratio in vitro and in vivo. Neuron. 1996; 17:1005–13. 10.1016/s0896-6273(00)80230-5 8938131

[14] Sun L, Zhou R, Yang G, Shi Y. Analysis of 138 pathogenic mutations in presenilin-1 on the in vitro production of Aβ42 and Aβ40 peptides by γ-secretase. Proc Natl Acad Sci U S A. 2017; 114:E476–85. 10.1073/pnas.1618657114 27930341 PMC5278480

[15] Spangenberg EE, Lee RJ, Najafi AR, Rice RA, Elmore MR, Blurton-Jones M, West BL, Green KN. Eliminating microglia in Alzheimer's mice prevents neuronal loss without modulating amyloid-β pathology. Brain. 2016; 139:1265–81. 10.1093/brain/aww016 26921617 PMC5006229

[16] Lyman M, Lloyd DG, Ji X, Vizcaychipi MP, Ma D. Neuroinflammation: the role and consequences. Neurosci Res. 2014; 79:1–12. 10.1016/j.neures.2013.10.004 24144733

[17] Yang J. Comprehensive description of protein structures using protein folding shape code. Proteins. 2008; 71:1497–518. 10.1002/prot.21932 18214949

[18] Yang J, Cheng WX, Zhao XF, Wu G, Sheng ST, Hu Q, Ge H, Qin Q, Jin X, Zhang L, Zhang P. Comprehensive folding variations for protein folding. Proteins. 2022; 90:1851–72. 10.1002/prot.26381 35514069

[19] Oakley H, Cole SL, Logan S, Maus E, Shao P, Craft J, Guillozet-Bongaarts A, Ohno M, Disterhoft J, Van Eldik L, Berry R, Vassar R. Intraneuronal beta-amyloid aggregates, neurodegeneration, and neuron loss in transgenic mice with five familial Alzheimer's disease mutations: potential factors in amyloid plaque formation. J Neurosci. 2006; 26:10129–40. 10.1523/JNEUROSCI.1202-06.2006 17021169 PMC6674618

[20] Mosher KI, Wyss-Coray T. Microglial dysfunction in brain aging and Alzheimer's disease. Biochem Pharmacol. 2014; 88:594–604. 10.1016/j.bcp.2014.01.008 24445162 PMC3972294

[21] Glass CK, Saijo K, Winner B, Marchetto MC, Gage FH. Mechanisms underlying inflammation in neurodegeneration. Cell. 2010; 140:918–34. 10.1016/j.cell.2010.02.016 20303880 PMC2873093

[22] Leng F, Edison P. Neuroinflammation and microglial activation in Alzheimer disease: where do we go from here? Nat Rev Neurol. 2021; 17:157–72. 10.1038/s41582-020-00435-y 33318676

[23] Liddelow SA, Guttenplan KA, Clarke LE, Bennett FC, Bohlen CJ, Schirmer L, Bennett ML, Münch AE, Chung WS, Peterson TC, Wilton DK, Frouin A, Napier BA, et al. Neurotoxic reactive astrocytes are induced by activated microglia. Nature. 2017; 541:481–7. 10.1038/nature21029 28099414 PMC5404890

[24] Colonna M, Butovsky O. Microglia Function in the Central Nervous System During Health and Neurodegeneration. Annu Rev Immunol. 2017; 35:441–68. 10.1146/annurev-immunol-051116-052358 28226226 PMC8167938

[25] Dantzer R, Konsman JP, Bluthé RM, Kelley KW. Neural and humoral pathways of communication from the immune system to the brain: parallel or convergent? Auton Neurosci. 2000; 85:60–5. 10.1016/S1566-0702(00)00220-4 11189027

[26] Copani A. The underexplored question of β-amyloid monomers. Eur J Pharmacol. 2017; 817:71–5. 10.1016/j.ejphar.2017.05.057 28577967

[27] Rosenblum WI. Why Alzheimer trials fail: removing soluble oligomeric beta amyloid is essential, inconsistent, and difficult. Neurobiol Aging. 2014; 35:969–74. 10.1016/j.neurobiolaging.2013.10.085 24210593

[28] Solomon B. Beta-amyloidbased immunotherapy as a treatment of Alzheimers disease. Drugs Today (Barc). 2007; 43:333–42. 10.1358/dot.2007.43.5.1062670 17724499

