# Supplementary material for: Impact of alternating amino acid sequences on beta-amyloid-induced neurotoxicity and neuroinflammation in Alzheimer's disease
Source: Aging (Albany NY). 2023 Oct 10;15(19):10580–92. doi: 10.18632/aging.205095 (PMC10599720; doi:10.18632/aging.205095)
Supplement: Supplementary Table 1 [file aging-15-205095-s001.pdf]

SUPPLEMENTARY TABLE

Supplementary Table 1. Primer sequences used for qRT-PCR in this work.

Gene name	Primer sequences (5′–3′)
Mouse-IL-1β	Forward: TGGCAACTGTTCCTG<br>Reverse: GGAAGCAGCCCTTCATCTTT
Mouse-TNFα	Forward: GCCTCTTCTCATTCCTGCTT<br>Reverse: TGGGAACTTCTCATCCCTTTG
Mouse-IFN-γ	Forward: CTCATGGCTGTTTCTGGCTG<br>Reverse: CCTTTTGCCAGTTCCTCCAG
Mouse-Rps18	Forward: GGATGTGAAGGATGGGAAGT<br>Reverse: CCCTCTATGGGCTCGAATTT
Mouse-IL-6	Forward: GACAAAGCCAGAGTCCTTCAGAGAG<br>Reverse: CTAGGTTTGCCGAGTAGATCTC
